# Home-based non-invasive brain stimulation for treatment-resistant depression

**DOI:** 10.1192/j.eurpsy.2025.1699

**Published:** 2025-08-26

**Authors:** R. Romero-Marín, S. López-Rodriguez, E. Buloz-Osorio, S. Fankhauser, N. Brault-Boixader, S. Delgado-Gallén, G. Albi-Villuendas, M. Cabello-Toscano, M. Urretavizcaya, M. Del Pino-Alonso, J. Solana-Sánchez, J. Camprodon, A. Pascual-Leone, D. Bartrés-Faz, D. Cappon, G. Cattaneo

**Affiliations:** 1 Research, Institut Guttmann; 2 Departament de medicina, Universitat Autonoma de Barcelona; 3 Institut d’Investigació Biomèdica de Bellvitge (IDIBELL); 4 Departament de medicina, Universitat de Barcelona, Barcelona, Spain; 5 Department of Psychiatry; 6 Department of Neurology, Harvard Medical School; 7 Hinda and Arthur Marcus Institute for Aging Research and Deanna and Sidney Wolk Center for Memory Health, Boston, United States; 8 Institut d’Investigacions Biomèdiques August Pi i Sunyer (IDIBAPS), Barcelona, Spain

## Abstract

**Introduction:**

Depression is a prevalent disease and 30% of affected patients are resistant to pharmacological treatment. Home-Based transcranial Direct Current Stimulation (HB-tDCS) has been proposed as a treatment option due to its low cost, minimal invasiveness, and scalability.

**Objectives:**

We present preliminary results on safety, feasibility and efficacy of a remotely supervised HB-tDCS intervention in patients with treatment-resistant depression.

**Methods:**

7 patients (5 women, age =55.67 ± 6.93) underwent a psychiatric evaluation, pre and post stimulation, that included the Montgomery-Asberg Depression Rating Scale (MADRS), the Beck Depression Inventory (BDI), and the Quick Inventory of Depressive Symptomatology (QIDS). HB-tDCS intervention consisted of 42 daily sessions administered through the Sooma tDCS™ device by a patients’ companion, trained by the research team. The anode was placed on the left prefrontal cortex, the cathode on the right prefrontal cortex, and 2mA current was delivered for 30 minutes.

After each session participants fulfilled an on-line survey for monitoring safety and feasibility.

**Results:**

86.73% of the sessions were completed. Due to impedance 7.84% of the sessions could not start on the first attempt, while 7.45% of the session were temporarily interrupted. Adverse effects included headaches (9.67%), sensations under electrodes (24.89%), and scalp dryness (7.88%).

We observed a significant reduction in depressive symptomatology as measured by the MADRS (-33.56%; t=-7.99, p<0.001). All patients showed partial response (>25%), and two a relevant response (>50%).

Self-reported scales indicated a reduction in symptomatology (QIDS:-21.66; t=-3.139, p=0.010; BDI:-13.92%; t=-1.780, p=0.063).

**Conclusions:**

In line with previous studies, these results indicate that HB-tDCS is a feasible, safe, and potentially effective intervention for treatment of resistant depression.

**Disclosure of Interest:**

None Declared

